# Synthesis, Characterization, *In Vitro* Cytotoxicity, and Apoptosis-Inducing Properties of Ruthenium(II) Complexes

**DOI:** 10.1371/journal.pone.0096082

**Published:** 2014-05-07

**Authors:** Li Xu, Nan-Jing Zhong, Yang-Yin Xie, Hong-Liang Huang, Guang-Bin Jiang, Yun-Jun Liu

**Affiliations:** 1 School of Chemistry and Chemical Engineering, Guangdong Pharmaceutical University, Zhongshan, P. R. China; 2 School of Food Science, Guangdong Pharmaceutical University, Zhongshan, P. R. China; 3 School of Pharmacy, Guangdong Pharmaceutical University, Guangzhou, P. R. China; 4 School of Life Science and Biopharmaceutical, Guangdong Pharmaceutical University, Guangzhou, P. R. China; Argonne National Laboratory, United States of America

## Abstract

Two new Ru(II) complexes, [Ru(bpy)_2_(FAMP)](ClO_4_)_2_ **1** and **2**, are synthesized and characterized by elemental analysis, electrospray mass spectrometry, and ^1^H nuclear magnetic resonance. The in vitro cytotoxicities and apoptosis-inducing properties of these complexes are extensively studied. Complexes **1** and **2** exhibit potent antiproliferative activities against a panel of human cancer cell lines. The cell cycle analysis shows that complexes **1** and **2** exhibit effective cell growth inhibition by triggering G0/G1 phase arrest and inducing apoptosis by mitochondrial dysfunction. The in vitro DNA binding properties of the two complexes are investigated by different spectrophotometric methods and viscosity measurements.

## Introduction

Since cisplatin and its platinum complexes were first used as anticancer agents successfully, more active transition metal complexes with better anticancer activity have been studied. Transition metal complexes have potential advantages, such as variable coordination numbers and geometries, accessible redox states, rich physicochemical properties, and extensive structural diversity, over common organic-based drugs. Therefore, the metal-based cancer drug is a focus of research in bioinorganic chemistry [1]–[6]. Cisplatin is widely used as an anticancer drug to treat many cancers, but severe side effects and acquired resistance caused by prolonged treatment have resulted in the search for alternatives to circumvent drug resistance [7]. With this goal, complexes based on several transition and non-transition metals have been investigated, among which Ru(II) has attracted much attention because of its variable oxidation states, selectivity for cancer cells, low toxicity, and ability to mimic iron when binding to biomolecules [8]. A number of ruthenium complexes display unique antitumor properties, and the treatment is not accompanied by major drug-related side effects [9]–[11]. such as NAMI-A and KP1019, have been used in clinical trials [12]–[13].

A large number of anthracene analogs have been reported as important DNA-intercalating agents with antitumor activity [14]–[15], including 9,10-anthracenedicarboxaldehyde bis[(4,5-dihydro-1*H* imidazol-2-yl)hydrazone]dihydrochloride, which has been evaluated in clinical phase I studies.^15^ However, the research of metal complexes based on analogs of anthracene is lacking. The development of metal complexes with bioactive molecules as ligands enables the synthesis of novel drugs that are more active and desirable than the ligands themselves [16]. Therefore, we reported the synthesis and characterization of two new, potential antiproliferative agents, [Ru(bpy)_2_(FAMP)](ClO_4_)_2_ **1** and [Ru(phen)_2_(FAMP)](ClO_4_)_2_ **2**, which were obtained by the combination of Ru(II) polypyridyl moieties with anthracene derivative. The antitumor activity, cell cycle arrest and apoptosis, DNA binding, and photocleavage properties of the two complexes were studied.

## Experimental Section

### Reagents and Materials

All reagents and solvents were purchased commercially and used without further purification unless specially noted. Ultrapure MilliQ water was used in all experiments. Calf thymus DNA (CT DNA) was obtained from the Sino-American Biotechnology Company. pBR 322 DNA was obtained from Shanghai Sangon Biological Engineering&Services Co., Ltd. Dimethyl sulfoxide (DMSO), RPMI 1640 (RPMI = Roswell Park Memorial Institute), 9, l0-bis(chloromethyl)anthracene and 2-nitro-propane were purchased from Sigma. RuCl_3_·xH_2_O was purchased from the Kunming Institution of Precious Metals. 1, 10-phenanthroline was obtained from the Guangzhou Chemical Reagent Factory. Cell lines of BEL-7402 (human hepatocellular carcinoma cell line), A549 (human lung adenocarcinoma epithelial cell line), MG-63 (human osteosarcoma cell line), and SKBR-3 (human breast cancer cell line) were purchased from the American Type Culture Collection. Agarose and ethidium bromide were obtained from Aldrich. Doubly distilled water was used to prepare buffers (5 mM Tris(hydroxymethylaminomethane)-HCl, 50 mM NaCl, pH = 7.2). A solution of calf thymus DNA in the buffer gave a ratio of UV absorbance at 260 and 280 nm of *ca*. 1.8∼1.9∶1, indicating that the DNA was sufficiently free of protein [17]. The DNA concentration per nucleotide was determined by absorption spectroscopy using the molar absorption coefficient (6600 M^–1^ cm^–1^) at 260 nm [18].

### Physical Measurements

Microanalyses (C, H, and N) were carried out with a Perkin-Elmer 240Q elemental analyzer. Fast atom bombardment (FAB) mass spectra were recorded on a VG ZAB-HS spectrometer in a 3-nitrobenzyl alcohol matrix. Electrospray ionization mass spectra (ES-MS) were recorded on a LCQ system (Finnigan MAT, USA) using methanol as mobile phase. The spray voltage, tube lens offset, capillary voltage and capillary temperature were set at 4.50 KV, 30.00 V, 23.00 V and 200°C, respectively, and the quoted *m/z* values are for the major peaks in the isotope distribution. ^1^H NMR spectra were recorded on a Varian-500 spectrometer with DMSO [D_6_] as solvent and tetramethylsilane (TMS) as an internal standard at 500 MHz at room temperature. UV/Vis spectra were recorded on a Perkin- Elmer Lambda 850 spectrophotometer and emission spectra were recorded on a Perkin-Elmer LS 55 spectrofluorophotometer at room temperature.

### Synthesis of the Ligand and Complexes

#### Synthesis of 2-(4-formylanthryl)imidazo-[4,5-f] [1], [10] phenanthroline (FAMP)

A mixture of 9, l0-Anthracenedicarboxaldehyde (0.35 g, 1.5 mmol) [19], 1,10-phenanthroline-5,6-dione (0.32 g, 1.5 mmol) [20], ammonium acetate (2.31 g, 30 mmol), and glacial acetic acid (30 cm^3^) was refluxed with stirring for 2 h. The cooled solution was then diluted with water and neutralized with concentrated aqueous ammonia. The precipitate was collected and purified by column chromatography on silica gel (60–100 mesh) with ethanol as eluent to give the compound as a yellow powder. Yield: 0.51 g, 80%. Anal. Calcd for C_28_H_16_N_4_O: C, 79.23; H, 3.80; N, 13.20. Found: C, 79.14; H, 3.91; N, 13.27%. FAB-MS: m/z = 425 (M+1).

#### Synthesis of [Ru(bpy)_2_(FAMP)](ClO_4_)_2_ (1)

A mixture of *cis*-[Ru(bpy)_2_Cl_2_]·2H_2_O (0.26 g, 0.5 mmol) [21] and FAMP (0.21 g, 0.5 mmol) in ethanol (20 cm^3^) was refluxed under argon for 8 h to give a clear red solution. Upon cooling, a red precipitate was obtained by dropwise addition of saturated aqueous NaClO_4_ solution. The crude product was purified by column chromatography on a neutral alumina with a mixture of MeCN-toluene (5∶1, v/v) as eluent. The red band was collected. The solvent was removed under reduced pressure and a red powder was obtained. Yield: 0.36 g, 70%. C_48_H_32_N_8_Cl_2_O_9_Ru: C, 55.61; H, 3.11; N, 10.81%; Found: C, 54.78; H, 3.21; N, 10.68%. ES-MS [CH_3_CN, m/z]: 837.2 ([M–2ClO_4_–H]^+^), 419.1 ([M–2ClO_4_]^2+^). ^1^H NMR (500 MHz, DMSO-d_6_): *δ* 14.16 (s, 1H), 13.08 (s, 1H), 9.08 (d, 2H, *J = *8.5 Hz), 8.94 (d, 2H, *J* = 9.0 Hz), 8.87 (d, 2H, *J* = 8.0 Hz), 8.83 (d, 4H, *J = *9.0 Hz), 8.76 (d, 2H, *J = *7.5 Hz), 8.51 (d, 2H, *J = *7.0 Hz), 8.21 (t, 2H, *J* = 6.0 Hz), 8.13 (d, 2H, *J = *6.5 Hz), 8.08 (t, 2H, *J* = 6.0 Hz), 7.97 (d, 2H, *J = *5.0 Hz), 7.84 (d, 4H, *J = *5.5 Hz), 7.57–7.61 (m, 2H), 7.35 (t, 2H, *J = *7.0 Hz).

#### Synthesis of [Ru(phen)_2_(FAMP)](ClO_4_)_2_ (2)

This complex was synthesized by an identical method as described for complex **1**, with *cis*-[Ru(phen)_2_Cl_2_]·2H_2_O [21] in place of *cis*-[Ru(bpy)_2_Cl_2_]·2H_2_O Yield: 0.35 g, 65%. C_52_H_32_N_8_Cl_2_O_9_Ru: C, 57.57; H, 2.97; N, 10.33%; Found: C, 57.65; H, 3.04; N, 10.28%. ES-MS [CH_3_CN, m/z]: 885.2 ([M–2ClO_4_–H]^+^), 443.1 ([M–2ClO_4_]^2+^). ^1^H NMR (500 MHz, DMSO-d_6_): *δ* 14.38 (s, 1H), 13.52 (s, 1H), 8.97 (d, 2H, *J = *8.5 Hz), 8.87 (d, 2H, *J* = 8.0 Hz), 8.76 (d, 2H, *J = *7.5 Hz), 8.67 (d, 2H, *J = *8.0 Hz), 8.62 (d, 2H, *J = *8.0 Hz), 8.54 (d, 2H, *J = *7.0 Hz), 8.31 (s, 4H), 8.21 (d, 2H, *J* = 5.5 Hz), 8.15 (d, 2H, *J = *6.5 Hz), 8.06 (d, 2H, *J* = 5.6 Hz), 7.94 (d, 2H, *J = *5.5 Hz), 7.84 (m, 2H), 7.75 (m, 4H).

#### Caution

Perchlorate salts of metal compounds with organic ligands are potentially explosive, and only small amounts of the material should be prepared and handled with great care.

### Cytotoxicity Assay in vitro

MTT (3-(4,5-dimethylthiazole)-2,5-diphenyltetraazolium bromide) assay procedures were used [22]. Cells were placed in 96-well microassay culture plates (8×10^3^ cells per well) and grown overnight at 37°C in a 5% CO_2_ incubator. Complexes were tested then added to the wells to achieve final concentrations ranging from 10^–6^ to 10^–4^ M. Control wells were prepared by addition of culture medium (100 µL). The plates were incubated at 37°C in a 5% CO_2_ incubator for 48 h. Upon completion of the incubation, stock MTT dye solution (20 µL, 5 mg·mL^–1^) was added to each well. After 4 h, buffer (100 µL) containing *N*, *N*-dimethylformamide (50%) and sodium dodecyl sulfate (20%) was added to solubilize the MTT formazan. The optical density of each well was then measured with a microplate spectrophotometer at a wavelength of 490 nm. The IC_50_ values were determined by plotting the percentage viability vs. concentration on a logarithmic graph and reading off the concentration at which 50% of cells remain viable relative to the control. Each experiment was repeated at least three times to obtain the mean values. Four different tumor cell lines were the subjects of this study: BEL-7402, A549, MG-63 and SKBR-3.

### Apoptosis Assay by Hoechst 33258 Staining Method

BEL-7402 cells were seeded onto chamber slides in six-well plates at a density of 2×10^5^ cells per well and incubated for 24 h. The cells were cultured in RPMI 1640 supplemented with 10% of fetal bovine serum (FBS) and incubated at 37°C and 5% CO_2_. The medium was removed and replaced with medium (final DMSO concentration, 1% v/v) containing the complex (12.5 µM) for 24 h. The medium was removed and the cells were washed with ice-cold PBS, and fixed with formalin (4%, w/v). Cell nuclei were counterstained with Hoechst 33258 (10 µg·mL^–1^ in PBS) for 10 min. Then the cells were observed and imaged by a fluorescence microscope (Nikon, Yokohama, Japan) with excitation at 350 nm and emission at 460 nm.

### Cell Cycle Arrest by Cytometric Analysis

BEL-7402 cells were seeded into six-well plates at a density of 2×10^5^ cells per well and incubated for 24 h. The cells were cultured in RPMI 1640 supplemented with fetal bovine serum (FBS, 10%) and incubated at 37°C and 5% CO_2_. The medium was removed and replaced with medium (final DMSO concentration, 1% v/v) containing complexes **1** and **2** (6.25 µM). After incubation for 24 h, the cell layer was trypsinized and washed with cold phosphate buffered saline (PBS) and fixed with 70% ethanol. The fixed cells are rinsed with PBS and then stained with the DNA fluorochrome PI in a solution containing Triton X-100 as well as RNase, keep 15 min at 37°C. Then the samples were analyzed with a FACSCalibur flow cytometer (Becton Dickinson & Co., Franklin Lakes, NJ). The number of cells analyzed for each sample was 10000 [23].

### Mitochondrial Membrane Potential Assay

Cells were treated for 24 h with complexes in 12-well plates and were then washed three times with cold PBS. The cells were then detached with trypsin-EDTA solution. Collected cells were incubated for 20 min with 1 µg·mL^–1^ of JC-1 (5,5′,6,6′-tetrachloro-1,1′,3,3′-tetraethylbenzimidazolcarbocyanine iodide) in culture medium at 37°C in the dark. Cells were immediately centrifuged to remove the supernatant. Cell pellets were suspended in PBS and then photographed under a microscope.

### DNA Binding and Photoactivated Cleavage

The DNA-binding and photoactivated cleavage experiments were performed at room temperature. Buffer A [5 mM tris(hydroxymethyl)aminomethane (Tris) hydrochloride, 50 mM NaCl, pH 7.0] was used for absorption titration, luminescence titration and viscosity measurements. Buffer B (50 mM Tris-HCl, 18 mM NaCl, pH 7.2) was used for DNA photocleavage experiments.

The absorption titrations of the complex in buffer were performed using a fixed concentration (10 µM) for complex to which increments of the DNA stock solution were added. The intrinsic binding constants *K*_b_, based on the absorption titration, were measured by monitoring the changes of absorption in the MLCT band with increasing concentration of DNA using the equation [24].

Thermal denaturation studies were carried out with a Perkin-Elmer Lambda 35 spectrophotometer equipped with a Peltier temperature-controlling programmer (±0.1°C). The melting temp (*T*m) was taken as themid-point of the hyperchromic transition. The melting curves were obtained by measuring the absorbance at 260 nm for solns of CT-DNA (100 µM ) in the absence and presence of the Ru^II^ complex (10 µM) as a function of the temp. The temp was scanned from 30 to 95°C at a speed of 1°C min^–1^. The data were presented as (*A* – *A_0_*)/(*A_f_* – *A_0_*) vs. temp., where *A*, *A_0_*, and *A_f_* are the observed, the initial, and the final absorbance at 260 nm, resp.

Viscosity measurements were carried out using an Ubbelodhe viscometer maintained at a constant temperature at 25.0 (±0.1) °C in a thermostatic bath. DNA samples approximately 200 base pairs in average length were prepared by sonicating in order to minimize complexities arising from DNA flexibility [25]. Flow time was measured with a digital stopwatch, and each sample was measured three times, and an average flow time was calculated. Relative viscosities for DNA in the presence and absence of complexes were calculated from the relation *η* = (*t*–*t^0^*)/*t^0^* [26]. Data were presented as (*η*/*η_0_*)^1/3^ versus binding ratio [27].

For the gel electrophoresis experiment, supercoiled pBR322 DNA (0.1 µg) was treated with the Ru(II) complexes in buffer B, and the solution was then irradiated at room temperature with a UV lamp (365 nm, 10 W). The samples were analyzed by electrophoresis for 1.5 h at 80 V on a 0.8% agarose gel in TBE (89 mM Tris-borate acid, 2 mM EDTA, pH = 8.3). The gel was stained with 1 µg/ml ethidium bromide and photographed on an Alpha Innotech IS–5500 fluorescence chemiluminescence and visible imaging system.

## Results and Discussion

### Synthesis and Characterization

An outline of the synthesis of complexes **1** and **2** is presented in Fig. S1. The ligand FAMP was obtained by condensation of 1,10-phenanthroline-5,6-dione with 9, l0-anthracenedicarboxaldehyde in refluxing glacial acetic acid containing ammonium acetate at a molar ratio of 1∶1. The complexes **1** and **2** were synthesized and high yield was obtained by the direct reaction of FAMP with appropriate precursor complexes in ethanol. The desired Ru(II) complexes were isolated as perchlorates and purified by column chromatography. In the ES–MS spectra of the Ru(II) complexes, the signals of [M-2ClO_4_-H]^+^ and [M-2ClO_4_]^2+^ were observed. The ES–MS results are provided in the experimental section. The measured molecular weights were consistent with expected values.

### Cytotoxicity Assays in vitro

The MTT assay procedures were used to evaluate the in vitro cytotoxicity of complexes **1** and **2** on BEL-7402, A549, MG-63, and SKBR-3 cell lines to determine their potential as anticancer agents. Cisplatin, the ligand FAMP and similar compound [Ru(phen)_2_(Hecip)](ClO_4_)_2_ were included for comparison, and were tested under identical conditions. The above cell lines were treated with different concentrations of complexes **1** and **2** for 48 h. The IC_50_ values for complexes **1** and **2** against these cell lines are summarized in Table 1. Based on the IC_50_ values, the newly synthesized complexes **1** and **2** were more active against SKBR-3 cells than against other cancer cells. The ligand FAMP showed less toxicity than complexes **1** and **2**. The IC_50_ values of complexes **1** and **2** toward BEL-7402 cells were lower than that of cisplatin, which indicates that complexes **1** and **2** show higher cytotoxic effect on BEL-7402 cells than cisplatin under identical conditions. Comparing the IC_50_ values, we also found that complex **1** was effective against BEL-7402 cells. Additionally, complex **2** shows higher cytotoxic activity than complex **1** against A549, MG-63, and SKBR-3 cell lines. The two complexes displayed different cytotoxic effect against different tumor cell lines. At the same time, the compound [Ru(phen)_2_(Hecip)](ClO_4_)_2_ shows lower IC_50_ values than complex **2** against BEL-7402, A549 and MG-63 cell lines. Based on the results, we speculate that the antitumor activity of complex may be related to the specific molecular shape of the complex and the chemical structure and nature of the inserted ligand.

**Table 1:** The IC_50_ values of tested compounds towards different cell lines.

Complex	IC_50_ (µM) BEL-7402	A549	MG-63	SKBR-3
**FAMP**	25.8±2.6	34.6±3.2	27.6±2.4	15.3±1.6
**1**	9.8±0.2	20.2±2.1	20.5±2.8	9.4±0.3
**2**	11.2±1.2	12.6±1.3	17.9±1.8	4.1±0.1
Ru(phen)_2_(Hecip)	10.6±0.4	9.6±0.5	15.2±2.3	8.6±0.2
cisplatin	19.2±2.2	20.6±3.1	6.7±2.6	−

### Apoptosis Assays with Hoechst 33258 Staining and Flow Cytometry

**Figure 1:**
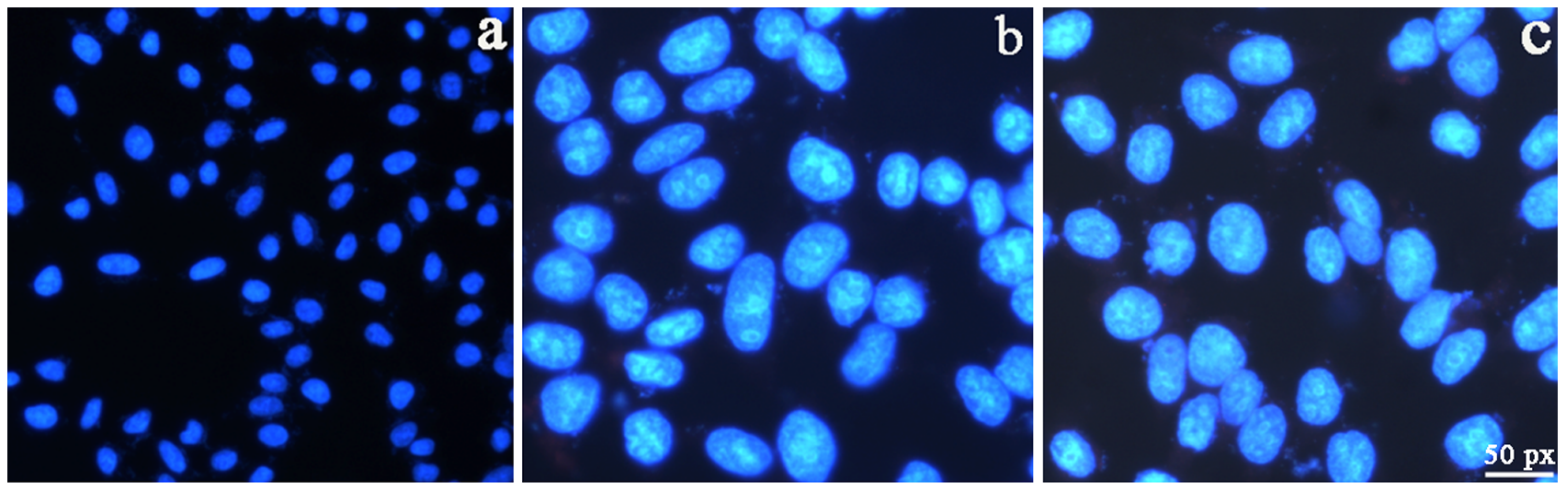
Hoechst 33258 staining of BEL-7402 cells for 24 h. a) control, b and c treated with 12.5 µM complexes **1** and **2**, respectively.

To determine whether or not complexes **1** and **2** induced chromatin condensation and fragmentation, which are recognized morphological features of apoptosis, BEL-7402 cell line was treated with complexes **1** and **2** at 12.5 µM for 24 h. As shown in Fig. 1, green apoptotic cells showing apoptosis characteristics such as nuclear shrinkage, chromatin condensation were observed. Complexes **1** and **2** can induce apoptosis in BEL-7402 cells.

**Figure 2:**
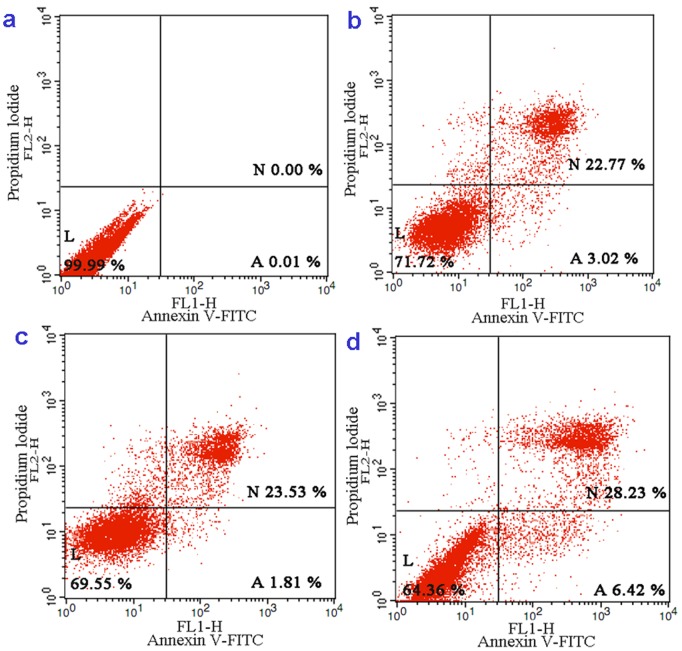
The percentage of Living (L), Necrotic (N) and Apoptotic (A) ruthenium complex-treated BEL-7402 cells as analyzed by FACS calibur flow cytometry. Control (a), 24 h exposed to 6.25 µM (b), 12.5 µM (c) and 25 µM (d) of complex **1**.

To determine the percentage of the necrotic or late apoptotic cells and early apoptotic BEL-7402 cells, the cells were treated with 6.25, 12.5 and 25 µM of complex **1** for 24 h. After treatment, cell apoptosis analyses were performed using flow cytometry. As shown in Fig. 2, at a concentration as low as 6.25 µM, 3.02% of the BEL-7402 cells were in the early apoptotic phase, whereas 22.77% of the cells were in the late apoptotic phase. However, at 12.5 and 25 µM, a total of 25.34% and 34.65% of the cells were undergoing apoptosis (early apoptotic+late apoptotic), respectively, whereas the untreated cells remained 100% viable. The cell death induced by complex **1** follows a pathway from the viable cells to the early apoptotic cells and then to the necrotic or late apoptotic cells. This pathway suggests that induced cell death occurs mainly through apoptosis. Furthermore, the effect increases significantly in a dose-dependent manner.

### Cell Cycle Arrest Studies

**Figure 3:**
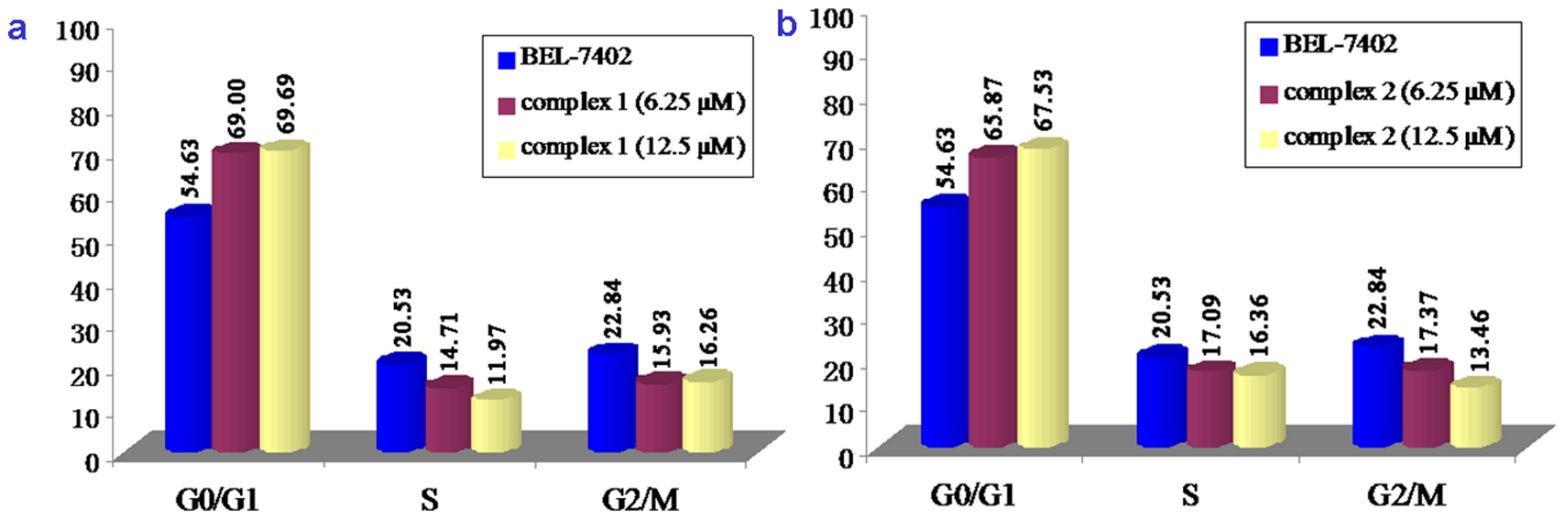
Cell cycle distribution of BEL-7402 cells exposure to different concentrations of complexes 1 (a) and 2 (b) for 24 h.

Flow cytometry was used to determine the effects of complexes **1** and **2** on the cell cycle progression of BEL-7402 cells. The cell cycle distribution after treating BEL-7402 cells with complexes **1** or **2** at 6.25 and 12.5 µM for 24 h is shown in Fig. 3. The percentage of cells at the G0/G1 phase showed an evident increase of 14.37% and 15.06% for complex **1** and 11.24% and 12.90% for complex **2** at 6.25 and 12.5 µM, respectively, which was accompanied by a corresponding reduction in the percentage of cells in the S and G2/M phases. Obviously, complex **1** caused more pronounced changes in the cell cycle than complex **2** in the G0/G1 phase under identical conditions. The antiproliferative mechanism induced by complexes **1** or **2** on BEL-7402 cells was G0/G1 phase arrest, and the changes in percentages at G0/G1 and S phases were concentration dependent.

### Mitochondrial Membrane Potential Detection

**Figure 4:**
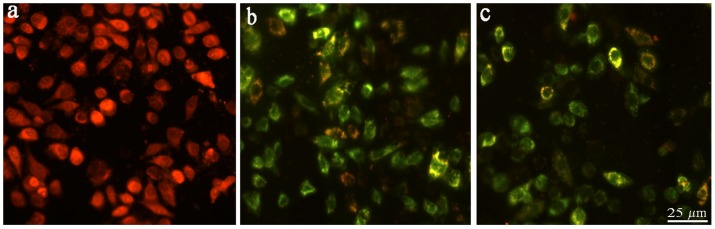
Assay of BEL-7402 cells mitochondrial membrane potential with JC-1 as fluorescence probe staining method. (a) control, after 24 h exposure to 12.5 µM of (b) complex **1** and (c) complex **2**.

JC-1 was used as a fluorescence probe in detecting the change of mitochondrial membrane potential induced by complexes **1** and **2**. At high mitochondrial membrane potential, JC-1 forms aggregates, which have a red fluorescence emission peak. At low mitochondrial membrane potential, JC-1 forms monomers, which emit a green fluorescence peak. As shown in Fig. 4, JC-1 exhibits a red fluorescence (JC-1 aggregates) with high mitochondrial membrane potential in the control. After exposure of BEL-7402 cells to 12.5 µM concentration of complexes **1** and **2** for 24 h, JC-1 showed green fluorescence (JC-1 monomers) corresponding to low mitochondrial membrane potential. The change from red to green fluorescence suggests the decrease of mitochondrial membrane potential in the presence of complexes **1** and **2**, which indicates that complexes **1** and **2** induced apoptosis in BEL-7402 cells through the mitochondrial signal transduction pathway.

### Electronic Absorption Spectra Studies

**Figure 5:**
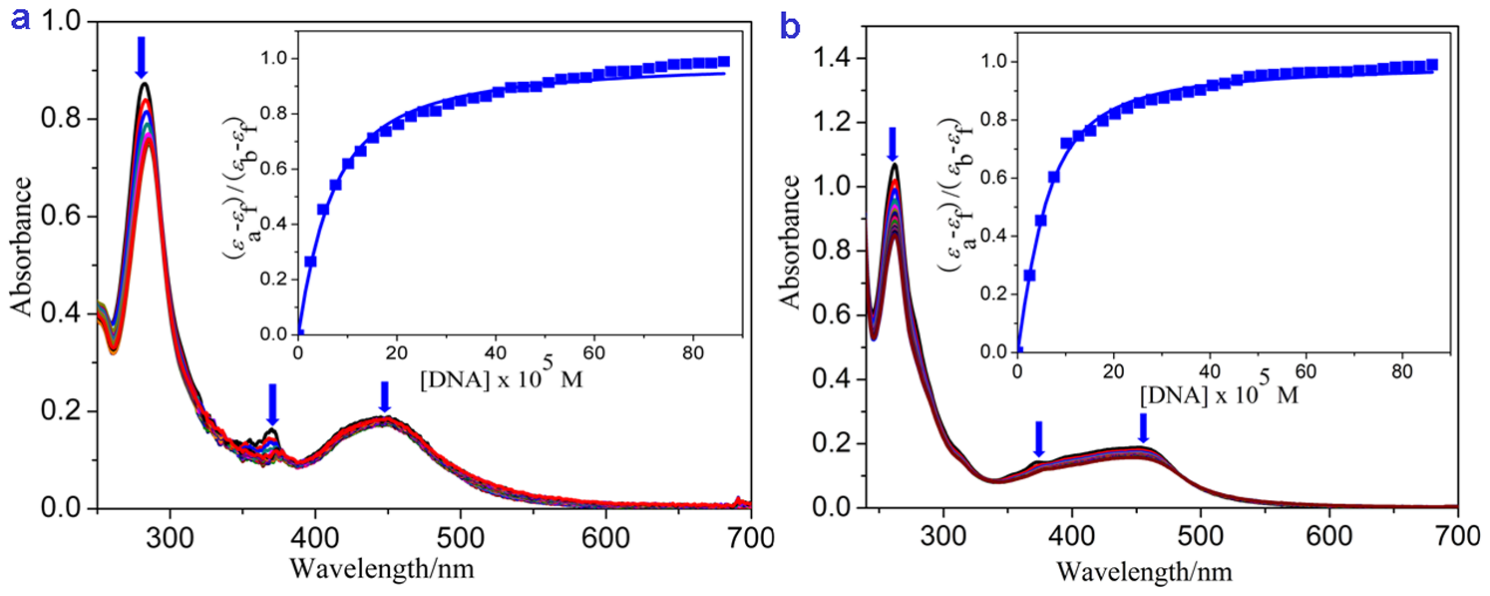
Absorption spectra of complexes in Tris-HCl buffer upon addition of CT-DNA in the presence of complexes a) 1 and b) 2. [Ru] = 10 µM. Arrow shows the absorbance change upon the increase of DNA concentration. Plots of (ε_a_-ε_f_)/(ε_b_-ε_f_) versus [DNA] for the titration of DNA with Ru(II) complexes.

The absorption spectra of complexes **1** and **2** in the presence of increasing amounts of calf thymus DNA (CT DNA) are shown in Fig. 5. The lowest energy bands at 451 nm for complex **1** and 447 nm for complex **2** were assigned to the metal-to-ligand charge transfer transition. The bands in the range of 300 nm to 400 nm were attributed to π–π* transitions. The bands below 300 nm were attributed to intraligand π–π* transitions. As the concentration of CT DNA increased, the MLCT transition band of complexes **1** and **2** exhibited hypochromism of 14.68% and 17.20%, respectively. The values of *K* for complexes **1** and **2** were 0.80±0.24×10^5^ M^-1^ (s = 1.88) and 1.70±0.25×10^5^ M^-1^ (s = 2.59), respectively. These values were smaller than those of [Ru(bpy)_2_(dppz)]^2+^ (4.9×10^6^ M^-1^) [28] and [Ru(phen)_2_(dppz)]^2+^ (5.1×10^6^ M^-1^) [28], but were comparable with those of Δ-[Ru(phen)_2_(dppz)]^2+^ (3.2×10^5^ M^-1^) and Λ-[Ru(phen)_2_(dppz)]^2+^ (1.7×10^5^ M^-1^) [29], and were much larger than those of other typical DNA intercalative Ru(II) complexes (1.1×10^4^ M^-1^ to 4.8×10^4^ M^-1^). The complexes intercalatively bind to DNA, which involves a strong stacking interaction between the aromatic chromophore and the base pairs of the DNA. Additionally, the larger DNA binding constant of complex **2** reflects its higher binding affinity and deeper intercalation than complex **1**.

### Luminescence Studies

**Figure 6:**
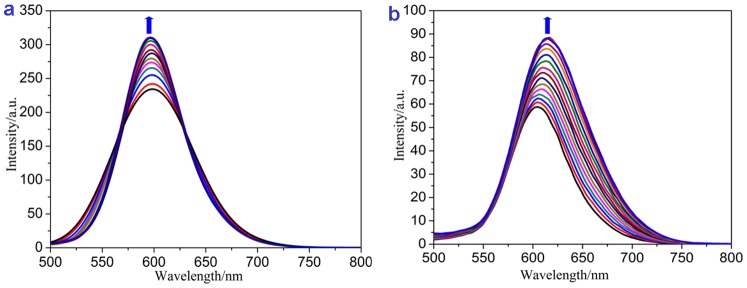
Emission spectra of complexes (a) 1 and (b) 2 in Tris–HCl buffer in the absence and presence of CT-DNA. Arrow shows the intensity change upon increasing DNA concentrations.

The absorption titrations and the luminescence studies confirm the satisfactory DNA binding abilities of complexes **1** and **2.** In the absence of DNA, complexes **1** and **2** can emit luminescence in Tris buffer at ambient temperature, with maximum luminescence appearing at 598 and 605 nm, respectively. With CT DNA addition, the emission intensities of complexes **1** and **2** increased to approximately 1.3 and 1.5 times the original intensities, respectively (Fig. 6), which implies that both complexes can interact with DNA and were protected by DNA efficiently. The increase in luminescence intensity is due to two factors: first, the hydrophobic environment inside the DNA helix reduces the accessibility of solvent water molecules to the complex; and second, the complex mobility is restricted at the binding site, leading to a decrease of the vibrational modes of relaxation. On the other hand, We found that the tendency of luminescent changes between complexes **1** and **2** is different. For complex **1**, the peak width became narrow with increasing concentration of DNA, However, The complex **2** displays a large red shift of the peak. The different luminescence behaviours of the complex-DNA system are mainly relevant to the affinity of the complex to DNA and the binding environment around the two molecules [30].

### DNA Thermal Denaturation Studies

**Figure 7:**
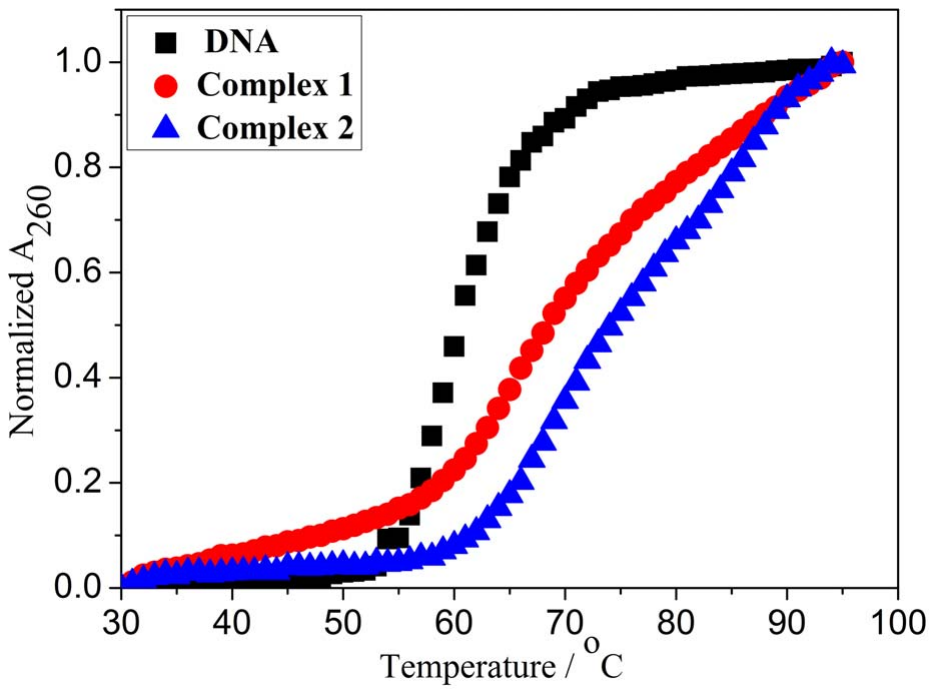
Thermal denaturation of CT-DNA in the absence (▪) and presence of complexes 1 (•) and 2 (▴). [Ru] = 10 µM, [DNA] = 100 µM.

DNA melting experiments can establish the extent of intercalation because the intercalation of the complex into DNA base pairs causes stabilization of base stacking, and therefore, raises the melting temperature of double-stranded DNA [31]. With increasing solution temperature, the double-stranded DNA gradually dissociates to single strands and generates a hyperchromic effect on the absorption spectra of the DNA bases (λ_max_ = 260 nm). To identify this transition process, the melting temperature *T*_m_, defined as the temperature when half of the total base pairs is unbonded, is usually introduced. A previous report [32] stated that the intercalation of a complex into DNA generally results in a considerable increase in the *T*_m_. The melting curves of CT DNA in the absence and presence of the complex are presented in Fig. 7. The thermal denaturation experiment performed for DNA in the absence of the Ru(II) complexes revealed a *T*_m_ of 60.5±0.2°C under our experimental conditions. The observed melting temperature in the presence of complexes **1** and **2** was 69.0±0.1°C and 74.2±0.2°C, respectively, at a concentration ratio of r = 0.10 (r = [Ru]/[DNA]). The Δ*T*_m_ values (8.5°C and 13.7°C) of complex **1**/**2**–DNA adducts are comparable to that observed for classical intercalators ethidium bromide (13°C, r = 0.10), Δ-[Ru(phen)_2_(dppz)]^2+^ (16°C, r = 1), and Δ-[Ru(phen)_2_(dppz)]^2+^ (5°C, r = 1) [29]. The results also suggest the intercalative binding mode of complexes **1** and **2** to DNA.

### Viscosity Measurements

**Figure 8:**
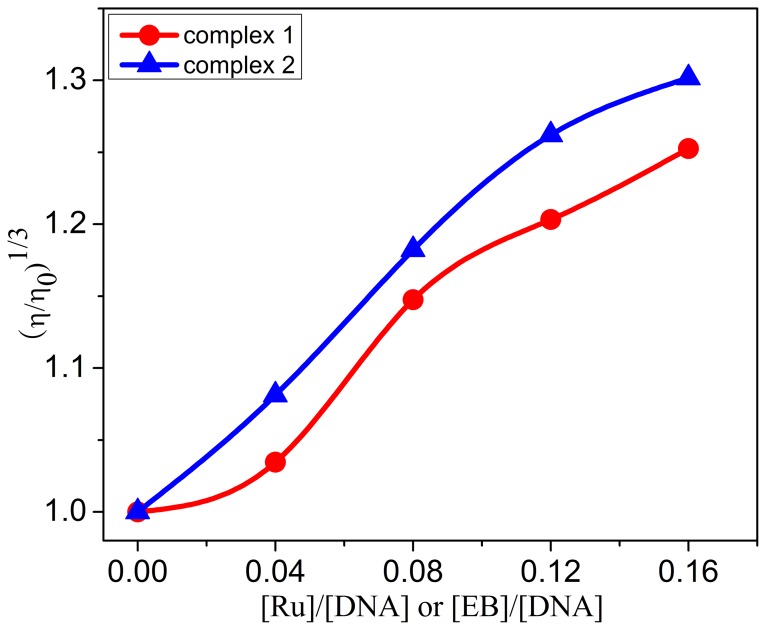
Effect of increasing amounts of complexes 1 (•) and 2 (▴) on the relative viscosity of calf thymus DNA at 25 (±0.1) °C [DNA] = 0.30 mM.

Relative viscosity measurements have been effective as a method for the assignment of the mode of binding compounds to DNA. In classical intercalation, the DNA helix lengthened as base pairs are separated to accommodate the binding ligand that increases DNA viscosity, whereas a partial, non-classical ligand intercalation causes a bend in the DNA helix reducing its effective length, and consequently, its viscosity. The viscosity studies provide a stronger argument for intercalation [33]. The effects of the complexes **1** and **2** on the viscosity of CT-DNA are shown in Fig. 8. As illustrated in Fig. 8, increasing the amount of the two complexes leads to a steady increase in the relative viscosity of CT DNA, which proves that the two complexes bind to DNA by classical intercalation. The increased degree of viscosity, which depends on the affinity of the complexes to the DNA, follows the order of complex **2**> complex **1**. The results are consistent with our conclusions.

### DNA Photocleavage

**Figure 9:**
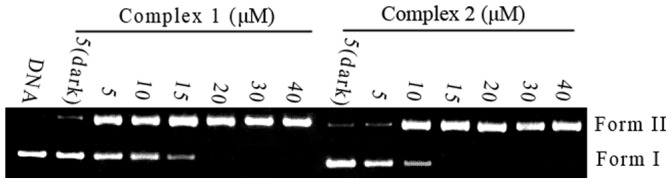
Photoactivated cleavage of pBR 322 DNA in the absence and presence of different concentrations of Ru(II) complexes 1 and 2 after irradiation at 365 nm for 30 min.

When circular plasmid DNA is subject to electrophoresis, relatively fast migration is observed for the intact supercoiled form (Form I). If scission occurs on one strand (nicked), the supercoiled form will relax to generate a slower-moving, open circular form (Form II) [34]. The cleavage of plasmid DNA in the absence or presence of complexes **1** and **2** was monitored by agarose gel electrophoresis. The DNA cleaving efficiencies of these complexes are shown in Fig. 9. No obvious DNA cleavage was observed in the controls that do not contain the complexes, or when the plasmid was incubated with the Ru^II^ complex in the dark. In the presence of different concentrations of the complexes, the Form I amount of pBR322 DNA decreased, whereas that of Form II increased. These results indicate that these complexes can effectively cleave pBR322 DNA upon irradiation.

## Conclusions

Two new Ru(II) complexes were synthesized and characterized. The cytotoxicity assay shows that complexes **1** and **2** can suppress tumor cells proliferation. Notably, the antiproliferative activities of complexes **1** and **2** are much higher than that of the ligand FAMP, suggesting that the significantly higher anticancer activity is due to the complexation with Ru(II) polypyridyl moieties. The apoptotic study indicates that complexes **1** and **2** can effectively induce apoptosis of BEL-7402 cells and inhibit the proliferation in the G0/G1 phase on BEL-7402 cells. Additionally, complexes **1** and **2** induce a decrease of the mitochondrial membrane potential. The complexes induce apoptosis of BEL-7402 cells through the mitochondrial signal transduction pathway. The DNA-binding behavior show that the two complexes interact with CT DNA through intercalative mode. Upon irradiation, these complexes can effectively cleave pBR322 DNA. These results will help in designing highly cytotoxic Ru(II) complexes and DNA-binding reagents.

## Supporting Information

Figure S1 Synthetic route of complexes **1** and **2**.
(TIF)
