# Supplementary figures and images for: Synthesis, Characterization, In Vitro Cytotoxicity, and Apoptosis-Inducing Properties of Ruthenium(II) Complexes
Source: PLoS One. 2014 May 7;9(5):e96082. doi: 10.1371/journal.pone.0096082 (PMC4013043; doi:10.1371/journal.pone.0096082)

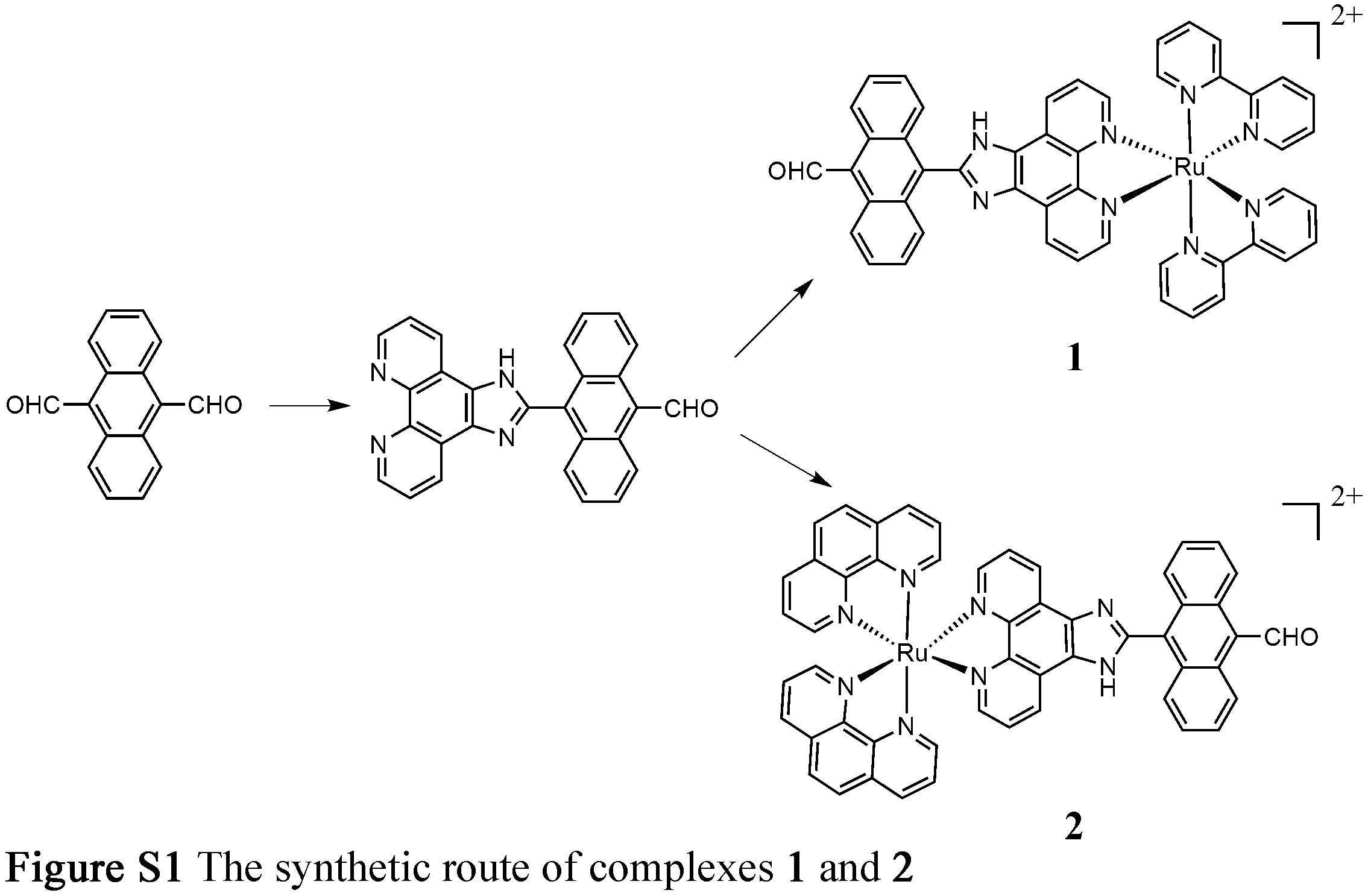

Supplement: Figure S1 — Synthetic route of complexes 1 and 2. (TIF) [file pone.0096082.s001.tif]
